# Global Dietary Patterns and Functional Gastrointestinal Disorders

**DOI:** 10.3390/children7100152

**Published:** 2020-09-27

**Authors:** Cara Hannah Axelrod, Miguel Saps

**Affiliations:** Department of Pediatric Gastroenterology, Hepatology, and Nutrition, University of Miami Miller School of Medicine, Miami, FL 33136, USA; msaps@med.miami.edu

**Keywords:** diet, culture, functional gastrointestinal disorders

## Abstract

Functional Gastrointestinal Disorders (FGIDs) are common. In the United States alone, approximately 25 million Americans are estimated to have at least one FGID. Nonpharmacological treatment options include psychological/behavioral approaches, and dietary interventions that can vary across countries. The aim of this review is to evaluate the available evidence for dietary interventions for the treatment of childhood FGIDs amongst various cultures and regions of the world. This review includes clinical trials of dietary therapies for the treatment of FGIDs in children posted on or before 13 July 2020 in PubMed. Overall, the consensus view suggests that the westernization of diets is linked to the development of FGIDs, and diets low in Fermentable Oligosaccharides, Disaccharides, Monosaccharides and Polyols (FODMAPs) may reduce abdominal symptoms. However, more work is needed to confirm these findings.

## 1. Introduction

Functional Gastrointestinal Disorders (FGIDs) are common. In the United States alone, approximately 25 million Americans are estimated to have at least one FGID [1]. Children with FGIDs report a reduced quality of life compared to healthy children [2]. Although the pathogenesis is multifactorial, psychosocial factors, diet and alterations in gut microbiota are thought to play a role [3].

Many patients with FGIDs report gastrointestinal symptoms to be meal related. The most commonly reported trigger for abdominal pain, bloating, diarrhea and distention in childhood is dairy [4]. The evidence for this is controversial and not sustained by the results of double blinded trials [5]. Other commonly reported triggers can include wheat products [6]. Consequently, dietary interventions excluding food containing dairy and gluten are frequently recommended by gastroenterologists [7].

One of the most commonly prescribed dietary interventions for Irritable Bowel Syndrome (IBS) is the low Fermentable Oligosaccharides, Disaccharides, Monosaccharides and Polyols (FODMAPs) diet. This diet was developed over 10 years ago in Australia by Monash University researchers. FODMAPs are a class of poorly digested carbohydrates that are fermented in the small bowel to produce gas and bloating [8]. Additionally, diarrhea-predominant IBS may be explained by the osmotic effect of unabsorbed FODMAPs in some cases [9]. FODMAPs are found in many different fruits, vegetables, legumes, cereals, honey, dairy products and sweeteners [8]. In the Western region, diets are largely carbohydrate-based [10]. Processed meat, pre-packaged foods, fried foods, refined grains and high-sugar drinks are highly prevalent [10].

Several studies have evaluated the efficacy of the low FODMAP diet for the treatment of FGIDs. Although there are successful adult trials, up to 50% of people who adhere to the diet are not relieved of their symptoms [11].

Treatment failure could be at least partially explained by the lack of dietitians to assist with counseling on the elimination (and reintroduction) of FODMAPs, inadequate access to resources, and lack of universal labeling laws [11]. Current labeling requirements do not require FODMAP content to be listed, and some foods that are considered to be low in FODMAPs have been found to contain large amounts of FODMAPs [12]. For example, Chumpitazi et al. demonstrated that gluten-free baked products and manufactured beverages had excessive amounts FODMAPs [12]. The FODMAP content of produce is also dependent on harvest time [13], which likely differs across different regions.

No study to date has explored dietary cultural aspects for childhood FGIDs. This subject is of great importance to study because dietary intakes vary across ethnic, religious and regional groups. We conducted a narrative review of the dietary practices of children in different areas of the world. This review aimed to highlight the dietary habits and dietary interventions for pediatric FGIDS among different regions of the world. Overall, FGIDs have not been differentiated in terms of their symptoms, diagnosis or dietary factors implicated. The information contained relates mainly to IBS and functional abdominal pain.

## 2. Data Sources and Study Selection

Clinical trials of dietary therapies for the treatment of FGIDs, posted on or before July 13, 2020, were searched for via PubMed. Search terms of ‘child’, ‘childhood’, ‘children’, ‘adolescents’, ‘abdominal pain’, ‘IBS’, ‘FGIDs’, ‘culture’, ‘continents’, ‘prevalence’, ‘Australia’, ‘United States’, ‘Africa’, ‘Europe’, ‘South America’ ‘Asia’, ‘gluten’ ‘nutrition’, ‘diet’, ‘dairy’, and ‘FODMAPs’ were used. Individual countries amongst each continent were also searched. Only trials published in English were included. Studies examining the role of dietary therapies for the treatment of organic diseases were excluded. Meta-analyses and case reports were also excluded, though the references were cross checked. Although the primary aim was to assess trials in children and adolescents, some adult studies were included to strengthen the review. As this review is focused on the impact of diet and FGID across regions, the data is presented by the country of origin. This study did not require ethical approval.

## 3. Summary of Findings

The relationship between diet and pediatric FGIDs has been explored by researchers internationally. We included 1 study with breastfeeding mothers and infants, 10 studies with children, 1 study with adolescents and 11 studies with adults. The majority of early studies identified individual isolated carbohydrates as triggers for symptoms, notably lactose and fructose. Recent studies have expanded to include all fermentable carbohydrates. A description of our findings, characterized by geographic area, is below (Table 1).

### 3.1. Australia

The typical Australian cuisine includes meat pies, chips and fish or hamburgers with beetroot. Common staples include Vegemite, a spread made from leftover brewer’s yeast extract and spices. Tim Tams chocolate biscuits and Fairy bread (bread with butter and sprinkles) are frequently consumed by children [14].

In 2000, Huang et al. reported that the consumption of specific carbohydrates (three or more servings of whole wheat bread or cereals per day) had a significant protective effect on reported abdominal pain in children in an Australian general practice [15]. Gibson et al. explored the idea that the ingestion of fermentable but poorly absorbed short chain carbohydrates may be a trigger for abdominal pain [16]. Gibson and his team hypothesized that limiting the intake of these carbohydrates would reduce abdominal symptoms in patients with IBS. Thus, a team of researchers at Monash University in Melbourne, Australia designed the low FODMAP diet [8].

In 2014, researchers aimed to determine if the low FODMAP diet could reduce abdominal symptoms, in a controlled cross-over study of adults with IBS. The authors found that gastrointestinal symptom scores were improved while on the low FODMAPs diet compared to the Australian diet [17]. In line with the previous study, Iacovou et al. showed that breastfeeding mothers who followed a low-FODMAP diet had reductions in crying-fussing durations of infants with colic [18]. Other treatment modalities, such as gut-directed hypnotherapy, were also shown to improve gastrointestinal symptoms similarly to the low FODMAP diet [19]. The fact that treatments aimed at the central nervous system also improve symptoms speaks of the role of the gut–brain axis in FGIDs.

### 3.2. North America

Similar to Australia, much of the work in the United State has focused on the malabsorption of carbohydrates. The Western diet is high in refined carbohydrates, red meat, sugar, salt, saturated fat, and high fructose corn syrup (HFCS). More specifically, in regards to HFCS, sweetened beverages and juice have approximately a 60:40 fructose-to-glucose ratio. Fructose consumption is a public health issue and has been linked to metabolic disease. In addition, prior research has demonstrated that spicy foods, cow’s milk and pizza are the top triggers of abdominal symptoms, and notably 2/3 contain dairy [20].

A 2003 study conducted in children with abdominal pain and lactose intolerance showed that consuming 12 g of lactose daily was associated with increased abdominal pain. There was a significant increase in abdominal pain experienced by study participants during the lactose ingestion period when compared to the lactose-free period, suggesting that a lactose-restricted diet could be beneficial to mitigate abdominal symptoms [21]. However, children who are not formally diagnosed with lactose intolerance often continue to complain of abdominal discomfort with dairy.

Considering that children in the US are large consumers of fruit juice-based HFCS beverages, Gomara et al. aimed to determine if fructose malabsorption was a significant problem in this age group in 2008. Thirty-two patients with abdominal pain were randomized to receive 1, 15 or 45 g fructose. The patients underwent breath hydrogen testing 3 h after ingestion. Of the 32 patients, 11 had positive test results, and 9 of 11 had an improvement of their symptoms by restricting fructose intake [22]. The results suggest two things. First, fructose malabsorption appears to be a significant problem in children. Second, reducing the dietary intake of fructose can be effective in reducing symptoms. Moreover, it is important to highlight the fact that in 2012, the US had the highest per capita consumption of HFCS [23]. Taken together, the available evidence could encourage practitioners in North America to discourage fructose-containing beverages.

In the last few years, much more information on FODMAP intolerance has become available. Several studies agree that FODMAP restriction can attenuate abdominal symptoms in childhood IBS. In a randomized, double blind, crossover trial conducted in Texas, 33 children with IBS had fewer daily abdominal pain episodes during the low FODMAPs period as compared to baseline [24]. Another study from Texas demonstrated that a low FODMAP diet reduces the frequency of abdominal pain in children with IBS [25]. A later investigation by the same author highlighted that fructans, found in wheat and onions, exacerbated several symptoms in childhood IBS [26]. This suggests that unlike wheat, common gluten-free vegetables, including onions, may represent a trigger for abdominal symptoms in certain patients with IBS.

No studies have evaluated different dietary interventions for childhood FGIDs in Canada, but prior investigations have implemented the low FODMAP diet in adults with success. A 2017 single blind study of adults with IBS demonstrated a reduction in symptoms for those on a low a FODMAP diet for 3 weeks [27]. This dietary intervention has shown a similar pattern of results in adult athletes as well. Runners with a history of exercise-related GI distress also found the low FODMAP diet to be a beneficial strategy for minimizing abdominal symptoms [28].

### 3.3. Africa and South America

The diet in Africa is mainly grain-based, with most people consuming less than one serving of fruits per day. Staple crops include maize, teff, cassava, yam, sweet potatoes and plantains. Beans, lentils and/or groundnuts are added to provide protein. Many of these foods are consumed in soup form with some green leafy vegetables.

Although the typical African diet is high in complex carbohydrates and other fiber-rich foods, urbanization has led to much more snacking on high fat/sugary foods in this region [29]. Epidemiologic studies that have shown that FGIDs are common to school children in Africa [30,31]; however, there is a scarcity of data about dietary interventions for FGIDs in the African continent. Thus, although many of the African foods are high in FODMAPs, and FGID prevalence is high, the association of diet and symptoms has not been investigated to date.

The diet in many countries in South America is described as beef-based, though it varies by region [32]. Salads and vegetables, including tomatoes, onions, lettuce, eggplants, squashes and zucchini, are common. Mate tea is a traditional drink of this region, and sorbet is a commonly consumed dessert [33]. Studies investigating dietary therapies for childhood FGIDs are lacking.

### 3.4. Europe

Diets vary amongst regions in Europe. The French diet includes cheeses, bread and wine, while the Italian and Sardinian diet includes vegetables, fruits, nuts, legumes and grains. Fish is consumed more frequently than red meat and dairy products [34].

Some work in Europe has investigated dietary risk factors for the development of FGIDs in childhood. More specifically, a study from Finland showed that compared to control children, fewer children with FGIDs had daily family dinner, and that children with FGID used vegetables, fruits and berries less often. Children with FGIDs consumed ice cream, soft drinks, and foods high in sucrose more frequently [35]. In Greece, a school-based, cross-sectional study showed that reduced fish and increased junk food consumption were related to a higher likelihood of developing functional abdominal pain disorders [36]. Another study concluded that good adherence to the Mediterranean diet is associated with a lower prevalence of FGIDs [37]. The Mediterranean diet is centered on whole grains, fruits, vegetables, beans, herbs, spices, nuts and healthy fats such as olive oil.

Adult studies conducted in Italy have shown promising results for the low FODMAP diet. Paduano et al. studied the effect of low-FODMAP, gluten-free and balanced diets in IBS patients. After 4 weeks of dietary intervention, symptom severity, bloating, abdominal pain and quality of life improved for all of them. However, 86% of the participants preferred the balanced diet compared to a 3% preference for the low FODMAP diet [38]. Similar findings were revealed by Cingolani et al. in a 2020 Italian cohort. IBS symptoms, including abdominal pain, bloating, stool consistency, severity of disease and quality of life, were improved with the low FODMAP diet [39]. Finally, researchers in Sweden compared the effect of a low FODMAP diet with a traditional IBS diet in 67 patients with IBS (33 low FODMAPs, 34 traditional IBS diet). The study demonstrated that a diet low in FODMAPs reduces IBS symptoms as well as traditional IBS dietary advice [40].

Studies in children are limited and come with mixed results. Two studies conducted in Germany found reduction in abdominal pain frequency and intensity with dietary fructose restriction in children [41,42]. Though the studies did not identify specific foods that were restricted, it is interesting to note that not only do apples represent the most popular fruit in Germany, but they also have a high fructose content [43]. In the Netherlands, Gijsbers et al. designed a study aimed at understanding whether fructose intolerance was responsible for recurrent abdominal pain in 220 children. The study failed to identify a link [44]. Despite their findings, the authors recognize that patients with ongoing intolerances should undergo repeated challenges. Some people react to as little as 5 g of fructose, and according to the Dutch National Food Consumption Survey 2007–2010, median intakes of fructose range from 56 to 61 g per day in adolescents [45].

### 3.5. Asia

Milk and dairy products, legumes/pulses, wheat, garlic, onion, cauliflower, cabbage and shallots are staples of many cuisines in Asian countries, and are all considered to be high FODMAP foods [46]. Despite this, the prevalence of FGIDs in adolescents is generally lower in eastern populations [47].

Several countries in Asia have studied the relation of foods to FGIDs. A 2019 observational study conducted in Japan suggested that the relationship between FODMAP-containing foods and gastrointestinal problems was not as well established as in Western studies. The authors found that wheat, cow’s milk, green beans, cauliflower, seaweed, soybeans and shellfish had the least significant relationship with abdominal symptoms, despite many of these foods being high in FODMAPs. In contrast, artificial sweeteners, margarine and tomato paste were most significantly related with abdominal symptoms [48]. In Malaysia, a small study on 16 adults with IBS found that only 50% of them were able to comply with the low FODMAP diet for 6 weeks. However, among those who were able to comply, there was an excellent symptom response—a 60% improvement in abdominal pain, bloating and flatulence [49].

In addition to FODMAPs, other dietary factors have been explored. A cross-sectional study of adults in Iran demonstrated that the consumption of spicy foods is directly associated with IBS [50]. In Taiwan, FGIDs were common among Taiwanese adolescents who choose fast-food consumption. Low fiber intake and an increased intake of frozen desserts in the diet was also linked to the development of these disorders [51].

## 4. Discussion

Diets vary amongst regions and cultures, and the role of diet in treating FGIDs is not universally accepted. There is little evidence for most of the treatments that are commonly prescribed. General blanket recommendations cannot be prescribed, and should be tailored to each individual.

Despite dairy being a commonly reported trigger, we found little evidence for its exclusion being effective in the treatment of FGIDs in children. In populations with well-known lactose intolerance as adults, such as Japan, only 6% have IBS [52]. If lactose intolerance was widely associated with IBS, we would expect a higher prevalence of IBS in areas where lactose intolerance is extremely frequent, and that is not the case for Japan, where almost 90% of adults have lactose intolerance [53]. However, although lactose intolerance is not the root of FGIDs, patients with visceral hypersensitivity who happen to have lactose intolerance will have a worsening of symptoms with lactose consumption.

Fructose is a monosaccharide (M’ in the FODMAP acronym) that, although being found naturally in many fruits and vegetables, is refined into HFCS and added to soft drinks and juices. This issue is a concern because even small fructose loads can overwhelm glucose transporters (glucose transporter 5 (GLUT5) and glucose transporter 2 (GLUT2)) that are required for fructose absorption [54]. In cases of fructose malabsorption, bacteria are fermented to produce short-chain fatty acids and gases that can result in pain, bloating, and changes in intestinal motility. Still, the link between fructose consumption and FGID is controversial. In the US, a study reported that fructose restriction can improve abdominal symptoms. This study included only 32 children [22]. Studies in Europe showed mixed results. This suggests that more work is needed to better understand the role of fructose in triggering FGIDs.

Similar to the case of lactose, subjects with hypersensitivity who consume high contents of fructose may develop severe symptoms, while similar consumption levels in subjects without visceral hypersensitivity may result in no or little symptoms. It is also important to remember that in cases where fructose ingestion is associated with glucose ingestion, the facilitated transport may increase absorption, and thus symptoms may be less likely to develop [55].

Though gluten-restricted diets are common in clinical practice [56], this review found little evidence for gluten elimination. Only one study in children showed the benefit of a double-blind challenge for children with non-celiac gluten sensitivity. The study showed that although this entity existed, it was rare [57]. The consequences of the unnecessary restriction of gluten in the diet should be considered, as the elimination of gluten may result in low fiber intake, which can negatively impact the gut microbiome and lead to constipation. Products that are gluten-free are also more expensive. A study found that gluten-free products are >180% more expensive than those containing gluten [58], and this issue may result in a financial burden to some families. In addition, children who cannot share meals with friends in school and outings may feel isolated or different, which can negatively affect their social integration.

We found several studies on FODMAPs in adults, yet very few in children. Only three trials in children have been published, all by Chumpitazi and his team. The results showed that FODMAP-restricted diets improve outcomes for children with FGIDs. However, there are several concerns that need to be taken into account regarding this dietary intervention. First, the FODMAP content of food is largely based on the Western literature, and may not necessarily be consistent in other parts of the world [46]. Fructose content, for example, can vary by location, harvest time and type. In addition, even amongst academic institutions there is confusion as to what constitutes a low FODMAP versus a high FODMAP food diet. McMeans et al. highlighted that >20% of the foods on three FODMAP food lists were in disagreement [59]. This suggests that there is a need to develop an individualized FODMAP content database per global region. Second, the international availability of low FODMAP foods for purchase is unclear. Third, the low FODMAP diet has been associated with deficiencies in iron and fiber, which could be problematic in growing children. Because of these potential limitations, this diet should only be used for limited periods of time and under the supervision of a dietitian. The extent to which that is possible in all countries is uncertain.

There are several limitations to this review. We only included papers published in English, and considering that this publication aimed to explore a global concern, the inclusion of other languages may have strengthened our review. Published studies may not represent the entire population of a particular country. Our study did not investigate religious practices that may have also influenced eating patterns. Lastly, it is difficult to draw associations between cross sectional dietary intake data and epidemiological FGID data.

In conclusion, despite the large global prevalence of abdominal symptoms in childhood, few randomized clinical trials have assessed the impact of dietary therapy on the treatment of FGIDs. These trials have largely been focused on the reduction of FODMAPs. Low FODMAP diets are supported by multiple adult studies, but adult data does not necessarily translate into children, and pediatric data is scarce. The lack of data is problematic for pediatricians and pediatric gastroenterologists who strive to base their plan of care on scientific evidence. Future studies are needed to validate global dietary therapies for the treatment of FGIDs in children. The integration of trained dietitians into all pediatric gastroenterology practices is recommended.

## Figures and Tables

**Table 1:** Summary of included studies.

Author	Title	Country	Populations Studied	GI Concern	Focus of Diets	Summary
Iacovou et al.	Randomised clinical trial: reducing the intake of dietary FODMAPs of breastfeeding mothers is associated with a greater improvement of the symptoms of infantile colic than for a typical diet.	Australia	Breastfeeding mothers and infants	Crying-fussing durations of infants with colic	FODMAPs	Maternal low-FODMAP diet was associated with less crying/ fussiness durations of infants with colic
Halmos et al.	A diet low in FODMAPs reduces symptoms of irritable bowel syndrome.	Australia	Adults	IBS	FODMAPs	A diet low in FODMAPs reduced functional gastrointestinal symptoms
Peters et al.	Randomised clinical trial: the efficacy of gut-directed hypnotherapy is similar to that of the low FODMAP diet for the treatment of irritable bowel syndrome.	Australia	Adults	IBS	FODMAPs	Effects of gut-directed hypnotherapy are similar to those of the low FODMAP diet
Huang et al.	Prevalence and pattern of childhood abdominal pain in an Australian general practice.	Australia	Children	Abdominal pain	Wholemeal bread or cereals	Children who consumed more than three servings of wholemeal bread or cereals each day were less likely to report abdominal pain
McIntosh et al.	FODMAPs alter symptoms and the metabolome of patients with IBS: a randomised controlled trial	Canada	Adults	IBS	FODMAPs	IBS symptoms were reduced in the low FODMAP diet group
Lis et al.	Low FODMAP: A Preliminary Strategy to Reduce Gastrointestinal Distress in Athletes.	Canada	Adults	Exercise-related GI distress	FODMAPs	Less gastrointestinal symptoms were reported on the low FODMAP diet
Wirth et al.	Positive or negative fructose breath test results do not predict response to fructose restricted diet in children with recurrent abdominal pain: results from a prospective randomized trial	Germany	Adults	Abdominal pain	Fructose	Fructose restricted diet in children and adolescents with recurrent abdominal pain may benefit abdominal pain symptoms
Wintermeyer et al.	Fructose Malabsorption in Children with Recurrent Abdominal Pain: Positive Effects of Dietary Treatment	Germany	Children	Abdominal pain	Fructose	Fructose restriction resulted in a decline of weekly pain frequency
Gijsbers et al.	Lactose and fructose malabsorption in children with recurrent abdominal pain: results of double-blinded testing.	Germany	Children	Abdominal pain	Lactose and fructose	Lactose intolerance nor fructose intolerance could be established as causes of abdominal pain
Agakidis et al.	Mediterranean Diet Adherence is Associated with Lower Prevalence of Functional Gastrointestinal Disorders in Children and Adolescents.	Greece	Children	Mixed FGIDs. 66% with functional constipation	Mediterranean Diet	Good adherence to the Mediterranean Diet is associated to lower prevalence of FGIDs
Chouliaras et al.	Dietary Habits and Abdominal Pain-related Functional Gastrointestinal Disorders: A School-based, Cross-sectional Analysis in Greek Children and Adolescents	Greece	Children	Abdominal pain	Fish/junk food	Reduced fish and increased junk food intake were related to more abdominal pain
Esmaillzadeh et al.	Consumption of spicy foods and the prevalence of irritable bowel syndrome.	Iran	Adults	IBS	Spicy food	Those consuming spicy foods greater or equal to 10 times per week more likely to have IBS compared to those who never consumed spicy foods
Cingolani et al.	Feasibility of Low Fermentable Oligosaccharide, Disaccharide, Monosaccharide, and Polyol Diet and Its Effects on Quality of Life in an Italian Cohort	Italy	Adults	IBS	FODMAPs	The low FODMAP diet lowered IBS severity
Paduano et al.	Effect of Three Diets (Low-FODMAP, Gluten-free and Balanced) on Irritable Bowel Syndrome Symptoms and Health-Related Quality of Life	Italy	Adults	IBS	FODMAPs, gluten-free, and balanced diets	All the three diets reduced symptom severity, bloating and abdominal pain, and improved quality of life
Böhn et al.	Diet low in FODMAPs Reduces Symptoms of Irritable Bowel Syndrome as Well as Traditional Dietary Advice: A Randomized Controlled Trial	Italy	Adults	IBS	FODMAPs	A diet low in FODMAPs, and traditional IBS dietary advice, reduced IBS symptoms
Wong et al.	Early experience with a low FODMAP diet in Asian patients with irritable bowel syndrome	Malaysia	Adults	IBS	FODMAPs	Improvement in IBS symptoms were reported in > 2/3 of patients
Shau et al.	Fast foods--are they a risk factor for functional gastrointestinal disorders?	Taiwan	Adolescents	Mixed FGIDs	Fast food	Fast-food consumption may contribute to a positive association with the development of FGIDs
McIntosh et al.	FODMAPs alter symptoms and the metabolome of patients with IBS: a randomised controlled trial	United States	Adults	IBS	FODMAPs	IBS symptoms were reduced in the low FODMAP diet group but not the high FODMAP group
Gremse et al.	Abdominal Pain Associated with Lactose Ingestion in Children with Lactose Intolerance. Clinical Pediatrics,	United States	Children	Abdominal pain	Lactose	There was a significant increase in abdominal pain during the lactose ingestion period compared to the lactose-free period
Chumpitazi et al.	Low FODMAPS diet ameliorates symptoms in children with irritable bowel syndrome: a double-blind, randomized crossover trial.	United States	Children	IBS	FODMAPs	A low FODMAPsdiet appeared to improve gastrointestinal symptoms within 48 hours in those with IBS
Chumpitazi et al.	Randomised clinical trial: gut microbiome biomarkers are associated with clinical response to a low FODMAP diet in children with the irritable bowel syndrome.	United States	Children	IBS	FODMAPs	A low FODMAP diet decreases abdominal pain frequency
Chumpitazi et al.	Fructans Exacerbate Symptoms in a Subset of Children With Irritable Bowel Syndrome.	United States	Children	IBS	Fructans	Fructans were found to exacerbate several gastrointestinal symptoms
Gomara et al.	Fructose intolerance in children presenting with abdominal pain.	United States	Children	Abdominal pain	Fructose	Fructose malabsorption is a problem in some children and managing dietary intake can be effective in reducing gastrointestinal symptoms

FGIDs = Functional Gastrointestinal Disorders; FODMAPs = Fermentable oligosaccharides, disaccharides, monosaccharides, and polyols; GI = Gastrointestinal; IBS = Irritable Bowel Syndrome
